# Origin and Emergence of Microglia in the CNS—An Interesting (Hi)story of an Eccentric Cell

**DOI:** 10.3390/cimb45030171

**Published:** 2023-03-22

**Authors:** Iasonas Dermitzakis, Maria Eleni Manthou, Soultana Meditskou, Marie-Ève Tremblay, Steven Petratos, Lida Zoupi, Marina Boziki, Evangelia Kesidou, Constantina Simeonidou, Paschalis Theotokis

**Affiliations:** 1 Department of Histology-Embryology, School of Medicine, Aristotle University of Thessaloniki, 54124 Thessaloniki, Greece; 2 Division of Medical Sciences, University of Victoria, Victoria, BC V8P 5C2, Canada; 3 Department of Neuroscience, Central Clinical School, Monash University, Melbourne, VIC 3004, Australia; 4 Centre for Discovery Brain Sciences & Simons Initiative for the Developing Brain, University of Edinburgh, Edinburgh EH8 9XD, UK; 5 Laboratory of Experimental Neurology and Neuroimmunology, Second Department of Neurology, AHEPA University Hospital, 54621 Thessaloniki, Greece; 6 Laboratory of Experimental Physiology, School of Medicine, Aristotle University of Thessaloniki, 54124 Thessaloniki, Greece

**Keywords:** microglia, origin, yolk sac, progeny, molecular cues, development

## Abstract

Microglia belong to tissue-resident macrophages of the central nervous system (CNS), representing the primary innate immune cells. This cell type constitutes ~7% of non-neuronal cells in the mammalian brain and has a variety of biological roles integral to homeostasis and pathophysiology from the late embryonic to adult brain. Its unique identity that distinguishes its “glial” features from tissue-resident macrophages resides in the fact that once entering the CNS, it is perennially exposed to a unique environment following the formation of the blood–brain barrier. Additionally, tissue-resident macrophage progenies derive from various peripheral sites that exhibit hematopoietic potential, and this has resulted in interpretation issues surrounding their origin. Intensive research endeavors have intended to track microglial progenitors during development and disease. The current review provides a corpus of recent evidence in an attempt to disentangle the birthplace of microglia from the progenitor state and underlies the molecular elements that drive microgliogenesis. Furthermore, it caters towards tracking the lineage spatiotemporally during embryonic development and outlining microglial repopulation in the mature CNS. This collection of data can potentially shed light on the therapeutic potential of microglia for CNS perturbations across various levels of severity.

## 1. Introduction

The central nervous system’s (CNS) principal innate immune cells are tissue-resident macrophages, which include microglia [1,2]. While microglia are the parenchymal brain macrophages, the perivascular, meningeal, and choroid plexus macrophages constitute the non-parenchymal tissue-resident macrophages of the CNS [3]. Microglia have a range of biological activities in both the developing and adult mammalian brain, although this population of cells makes up the lowest percentage of non-neuronal cells in the mammalian brain [4]. The release of mediators (e.g., trophic factors, cytokines) and phagocytosis are the two main mechanisms by which microglia shape brain development and perform key functions across life [5]. These microglial activities are implicated in developmental processes such as synaptic patterning, myelinogenesis, axonal dynamics, cell positioning, and survival [6]. In the adult brain, microglial activities are central to the regulation of acute and chronic immune responses and maintenance of CNS homeostasis through the removal of viruses, bacteria, and other foreign particles, but also cellular debris and synapses, mediation of neurogenesis following CNS injury, and protection of neural tissue [7,8,9]. However, microglia as innate immune cells are sensitive to chronic inflammation, which can impair their beneficial functions and participate in the etiology of protracted neurodegenerative diseases such as Parkinson’s and Alzheimer’s diseases, multiple sclerosis, and amyotrophic lateral sclerosis (ALS) [10,11,12].

The term microglia—micro (small) and glia (glue)—was first introduced in 1919 by Pío del Río Hortega, who proposed that microglia adopt a malleable morphology, transforming from a resting to an activated state during disease exhibiting phagocytic properties [13]. This view was recently considered too simplistic, as microglia can adopt a wide variety of morphological and functional states [5]. Under normal physiological conditions, microglia display a ramified morphology with multiple branches and processes constantly surveilling the CNS parenchyma. Inflammatory stimuli can change microglial morphology, for instance converting microglia from a ramified to an amoeboid form characterized by an enlarged cell body and retracted processes. In contrast to the ramified state, amoeboid microglia with an amoeboid morphology are generally considered to exhibit a high phagocytic and proinflammatory phenotype. The activated microglial cells were previously categorized as: classical (M1) or alternative (M2), corresponding to either a proinflammatory and neurotoxic state or an anti-inflammatory state, respectively. However, it is now suggested that the M1/M2 phenotypes are not representative of in vivo microglia states because microglia rarely appear with a distinct M1 or M2 phenotype [5,14].

Tissue-resident macrophages are present in the CNS and across different organs, such as osteoclasts in bone, intestinal macrophages in the gastrointestinal tract, Kupffer cells in liver, alveolar macrophages in lungs, and Langerhans cells in skin [1]. Microglial cells are unique tissue-resident macrophages that differ from their hematogenous origins due to their surrounding environment, which is immune-privileged owing to the formation of the blood–brain barrier (BBB). The timing of BBB formation is vital for the invasion of microglia progenitors during embryonic development. Many studies have delineated that closure is vitally important at specific embryonic days. The permeability of BBB was found to decrease for large molecules from E12.5, and it became impermeable to small molecules as early as E14.5 [15]. This tight regulation showcases the importance of CNS master regulator elements to protect the central environment from pathogens and other harmful agents.

Each tissue-resident macrophage type has a distinct embryonic origin, as their progenitors derive from different waves of hemopoiesis. Consequently, the understanding of embryonic hematopoiesis is vital for delineating the microglial origin. Regarding hematopoiesis in embryonic life, three waves have been described in *mice*. The primitive hematopoiesis starts at E7.5 in the yolk sac (YS), generating primitive erythroid, megakaryocyte, and macrophage progenitors such as early c-MYB-independent erythro-myeloid precursors (EMPs). The second hematopoietic wave, called transient definitive hematopoiesis, originates from YS hemogenic endothelium, giving rise to late c-MYB-dependent EMPs at E8.25 and progenitors with lymphoid potentials at E9, which additionally emerge from the developing aorta-gonad-mesonephros (AGM) region. The definitive hematopoiesis occurs at E10.5 with the generation of hematopoietic stem cells (HSCs) that originate from the embryonic AGM region, colonizing slightly later the fetal liver [16,17,18,19].

The present review aims to define existing data on the origin of microglia because there has been controversy over their ontogeny. The developmental milestones that are being covered herein are the primary cues that direct microgliogenesis. The ontogeny of microglia is investigated thoroughly, as it is of prime importance considering that this cell type is involved in the pathogenesis of many diseases and is presented as a target for therapies that are being developed to control the associated phenotypes.

## 2. Discovery and Ontogeny of Microglia

**Figure 1:**
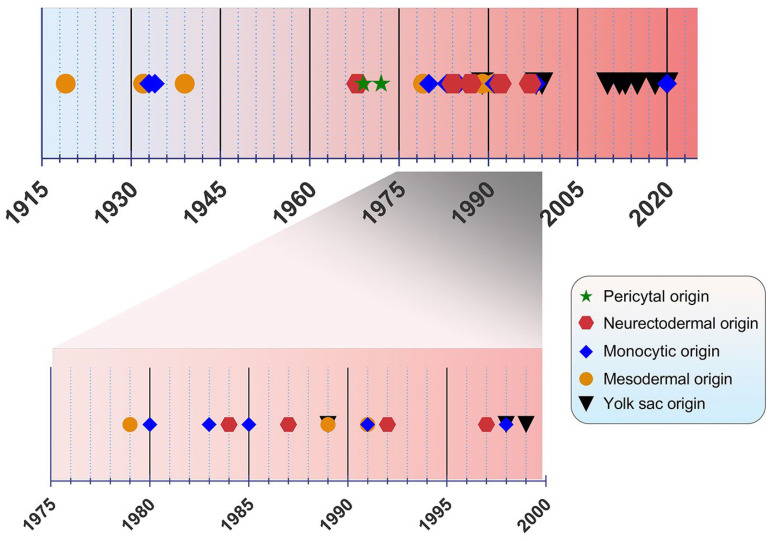
Timeline view of microglial origin since their discovery by Río Hortega.

The origin of microglia has been a debated topic for years. In the past, four main origin concepts have been proposed as a source of microglia: (i) the mesodermal-associated mater elements, (ii) the neuroectodermal matrix cells, (iii) the pericytes, and (iv) the invasion of monocytes especially during early development (Figure 1) [20]. Río Hortega was hailed as the “Father of Microglia”, because their discovery supported the mesodermal origin, after observing the invasion of the pial elements within the CNS parenchyma [13,21]. Using comparable staining methods, John Kershman agreed on the mesenchymal origin of microglia, which were found to be genetically related to histiocytes, a stationary phagocytic cell present in connective tissue [22]. With reference to Boya et al., the meningeal envelope was proposed to be the source of microglia, which sustains a mesodermal nature in agreement with the classical experiments by Río Hortega [23,24]. Later, the theory of multiple mesodermal sources of microglia depending on time and localization was posited [25]. However, another study proposed vascular pericytes as the parent cells of microglia [26,27]. The first reports on the monocytic origin of microglia came to the fore in 1933 and 1934 from Santha and Juba, respectively, who hypothesized that ramified microglia originated from circulating monocytes because the initial appearance of these cells coincided with the vascularization of the brain [28,29].

In the following decades, many researchers accepted this view, demonstrating microglial monocytic identity when investigating their origin [30,31,32,33], while others rejected the possibility that microglia are derived from mononuclear blood cells [34]. In 1968, autoradiography experiments performed with tritiated thymidine were conducted in adult *rats*, showing that cells of the subependymal layer give rise to a number of glial cell types, such as astrocytes and microglia, offering a different perspective regarding microglial origin [35]. The neuroectodermal origin was also supported by Kitamura et al., implying that glioblasts are the source of both astrocytes and microglia in *mice* [36]. It was also proposed that microglia and astroglia have a common progenitor cell developing from neuroepithelial cells [37]. Performing non-radioactive in situ hybridization and immunoperoxidase techniques, only a small population of microglia were found to be derived from bone marrow progenitors, because most of the cells were shown to be generated from locally residing precursors with a neuroectodermal ontogeny [38]. The non-monocytic origin of microglia favored by Schelper and Adrian implicated that these cells are CNS intrinsic ones, enforcing the above theory of a neuroectodermal origin [39]. This perception was also put forward by other researchers, but began to lose ground from the 2000’s onwards. [40,41].

The view of the origin of microglia from the YS was first introduced in 1989 [42]. A nucleoside diphosphatase histochemical study was conducted to evaluate the distribution of microglia in the developing *human* CNS, implying that mesenchymal cells with haemopoietic potential migrate into neural tissues and then give rise to cells resembling microglia [43]. Likewise, primitive macrophages of YS were found to be derived from fetal macrophages before the appearance of pro-monocytes/monocytes colonizing the embryonic tissues in *mice* [42]. In an *avian* model, microglia precursors were demonstrated to invade neural tissue from the pial surface and proliferated inside the CNS, indicating that their penetration through the embryonic CNS vessels is not possible [44]. However, a *human* embryogenesis study using lectin^+^ and CD68^+^ markers revealed two populations of microglia, indicating two different potential origins, specifically from the YS and bone marrow. Different routes of entry were also proposed: one through the mesenchyme and the other via the blood circulation [45]. Alliot et al., aiming to delineate the origin of microglia in *mice*, detected these cells in the brain from Ε8 being derived from YS progenitors, which proliferate in situ [46].

The YS origin of microglia was confirmed by Ginhoux et al. by performing a fate mapping analysis in *mice* and showing that YS primitive myeloid progenitors generated before E7.5 can contribute to the CNS microglial population [47]. Moreover, in this study, RUNX1^+^ YS progenitors were found to migrate into the brain through blood vessels between E8.5 and E9.5 [47]. The YS origin was further supported by identifying the transcription factor MYB, which is required for the development of HSCs as well as CD11b^high^ monocytes and macrophages [48], contrary to YS-derived macrophages, which are the potential precursors of CNS microglia [49]. Specifically, primitive c-kit^+^ EMPs detected from E8 in the YS were proposed to serve as the precursors of microglia in *mice* [50]. As the progenitors of microglia were identified to be the EMPs of YS, the vast majority of other tissue-resident macrophages arise from fetal monocytes that derive from late c-MYB^+^ EMPs of the YS [51]. The HSC-derived hematopoiesis that takes place for monocytes at E14.5 and granulocytes at E16.5 in *mice* advocates that these progenitors only seldom replace parenchymal microglia, which mainly emanates from CSF-1R^+^ EMPs. [52]. This view was re-evaluated by Sheng et al., who developed the Kit^MercreMer^ fate mapping *mouse* strain and suggested that all resident-tissue macrophages, except microglia and Langerhans cells of the epidermis, are derived from HSCs [53].

In 2018, De et al. identified two distinct microglial cell populations, namely canonical (non-HOXB8) and HOXB8 microglia using a transgenic strategy, fluorescence-activated cell sorting technique in YS and qRT-PCR in HOXB8 cells in the different hematopoietic tissues [54]. The HOXB8 population was suggested to be derived from the second wave of YS hematopoiesis populating the AGM and fetal liver. Besides the YS, an additional source of microglia was proposed by Fehrenbach et al., who considered the definitive hemopoiesis as responsible for microglial development and recruitment to the *mouse* CNS, especially at the post-YS phase [55]. Besides parenchymal microglia, a genetic distinct population of macrophages was identified, namely the border-associated macrophages (BAMs) residing among the meninges, choroid plexus, and perivascular spaces. Like microglia, these cells are generated by early EMPs; however, microglia require TGF-β for their development, whereas BAMs are TGF-β-independent. Additionally, in the *mouse* YS, two distinctive primitive populations were observed: the CD206^−^ and CD206^+^ macrophages. The differentiation of these populations after their final colonization is mediated by environmental drivers [56]. Interestingly, tamoxifen dosing in CCR2-CreER transgenic *mice* suggested that not only YS EMPs, but also fetal HSC-derived monocytes participate in the generation of IBA1^+^TMEM119^+^P2RY12^+^ parenchymal microglia, IBA1^+^, and isolectin^+^ BAMs in the *mouse* brain [57]. Lastly, a recent study in eight aborted *human* embryos proposed that tissue-resident macrophages development is very similar to other mammalian species, highlighting the presence of two distinct waves of YS-derived macrophages. Specifically for microglia, they were found to be derived from the early first wave along with a minor contribution from the second one [58].

To recapitulate, the YS is the main site of microglial origin. The suggested microglial progenitors in *mice* are the early, c-ΜΥΒ-independent, CSF-1R^+^ EMPs of the YS. However, the definite nomenclature of the progenitors and the confirmation in *human* models are still under consideration.

## 3. Molecular Cues Orchestrating Microgliogenesis

Upon birth, the phenotype of microglia corresponds to an amoeboid shape, phagocytically and mitotically active, while in later developmental stages, microglia become ramified. The RUNX1, a transcription factor expressed during the first two postnatal weeks at the forebrain by amoeboid microglia, downregulates the proliferation of these cells and assists in their transformation towards a ramified morphology [59]. During embryonic development, RUNX1 controls the expression of the transcription factor PU.1 [60]. In *Irf8*-deficient YS, the number of A1 cells (CD45^+^ c-kit^lo^ CX_3_CR1^−^ immature cells) remained unchanged, while the A2 population (CD45^+^ c-kit^−^ CX_3_CR1^+^ cells) decreased [50]. Additionally, *Pu.1* deficiency provoked an impairment of A1 and A2 progenitors. From A2 cells, microglia were generated and expanded in the developing brain under the influence of specific matrix metalloproteinases, such as MMP-9 and MMP-8. Factors such as MYB, BATF3, ID2, Klf4, and NR4A1 were not necessary for the development of microglia from their progenitors [50,61]. While PU.1 was essential for terminal myeloid differentiation, early myeloid genes such as *Gm-csfr*, *G-csfr*, and *Mpo* were maintained in *Pu.1*^-/-^ embryos, whereas myeloid genes associated with terminal differentiation (etc*. Cd11b*, *Cd64*, and *M-csfr*) were found to be impaired [62].

The CSF-1R is a vital receptor for microglial cell development expressed on YS macrophages and microglia at E9.5 and throughout brain development. In contrast to many tissue macrophages, adult microglia can still be replenished, albeit at reduced levels in *Csf-1*^op/op^ *mice*. Although the microglia presented—even in small amounts—in a null mutation model of the *Csf-1* in *Csf-1*^op/op^ *mice*, microglia were fully depleted in *mice* lacking CSF-1R [47]. This was a strong clue that a second ligand of CSF-1R, namely the IL-34, was implicated in microgliogenesis. As the microglial phenotype in *Csf-1r*^-/-^ *mice* was more severe than that observed in *Csf-1*^op/op^ *mice*, it was evident that IL-34 plays a significant role in the regulation of microglial homeostasis. Its mRNA expression in the brain is also significantly higher than that of CSF-1 during early postnatal development [47]. In addition, in *il-34*- and *csf-1ra*-deficient *zebrafish larva*, the migration and colonization of CNS by embryonic macrophages was impaired, indicating a role for the Il34-Csf1ra pathway during microglial cell expansion throughout the CNS [63].

**Figure 2:**
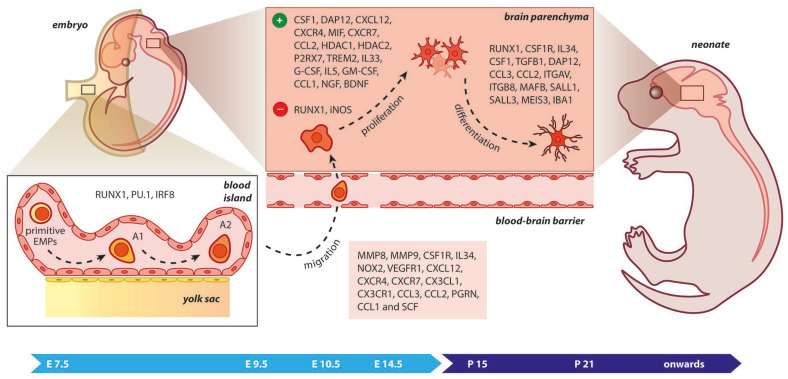
Microgliogenesis at a glance. The primitive erythro-myeloid progenitors (EMPs; early, c−MYB−independent, CSF−1R^+^ EMPs) arise from the yolk sac (YS) as early as embryonic day 7.5 (E7.5). These cells give rise to CD45^+^ c−kit_lo_ CX_3_CR1^−^ immature (A1) cells that develop into CD45^+^ c−kit^−^ CX_3_CR1^+^ (A2) cells. The early differentiation of microglial progenitors is regulated by the expression of RUNX1, PU.1, and IRF8. The invasion of progenitors into the neural tube begins at E9.5 through blood circulation and is followed by proliferation and terminal differentiation. As the blood–brain barrier becomes impermeable to small molecules at E14.5, the microglia invasion may be prevented. The transformation of immature microglia into ramified (mature form) occurs between the second and third postnatal weeks. The migration, proliferation, and terminal differentiation of microglia are also orchestrated from the depicted molecular cues. Light blue arrow timeline represents prenatal period, dark blue arrow timeline refers to postnatal days.

Microglia require TGF-β signaling to be maintained in their surveillant state, but not for their survival. The absence of TGF-β1 was found to have an impact on microglial development from E14.5, but not on microglial progenitors at E10.5 [64,65]. Other transcription factors that include SALL1, SALL3, and MEIS3 are involved in the specification of tissue-resident macrophages during organogenesis and ensure microglial function [66]. In fact, when the SALL1 locus was inducibly inactivated, microglial cells transformed from a ramified morphology to pro-inflammatory deregulating tissue homeostasis [64]. A sharp decline in the number of microglial cells was observed in postnatal *Dap12*-deficient *mice* that was comparable to the in vitro impairment of microglial cell differentiation [67]. This may be due to M-CSF’s role in inducing microglial proliferation and survival via a pathway requiring DAP12 and β-catenin. However, another study showed that microglial populations remained unaffected in *Dap12*-deficient *mice* similar to young (embryonic and early postnatal) wild-type *mice*, while a reduction in their numbers was observed in specific CNS regions of deficient adult *mice* [50,68]. In *Nox2* gene deficiency, treatment with apocynin, which is a NOX2 inhibitor, or impairment of the VEGFR1 kinase resulted in microglia that could not migrate efficiently into the caudal subventricular zone (SVZ) of the cerebral cortex, suggesting that chemotaxis of microglia was under the influence of NOX2 and VEGFR1 activation (Figure 2) [69].

The depletion of *Cxcl12* seems to block microglial cell invasion into the SVZ, whereas the ectopic *Cxcl12* expression or pharmacological impairment of CXCR4 demonstrated that the CXCL12/CXCR4 signaling is involved in microglial cell recruitment assisting cortical development. In the same context, cell death occurring in the developing forebrain stimulates microglial cell proliferation mediated via MIF activation [70]. Treatment with CXCL12 activates Erk1/2 and Akt signaling, which are necessary for microglial proliferation mediated by CXCL12. Similarly, Erk1/2 signaling was found to be important for CXCL12-depedent migration of microglial populations. Pharmacological blockade of CXCR4 or CXCR7 induced a decline in CXCL12-mediated proliferation and migration of microglial cells, suggesting that CXCR4 and CXCR7 form a receptor unit for CXCL12 in the *rodent* microglia required for the aforementioned developmental processes, both in vitro and in vivo [71]. Furthermore, CX3CL1/CX3CR1 signaling may regulate microglial invasion within CNS parenchyma during postnatal life [72]. Interestingly, the transformation of microglia from an amoeboid to a ramified morphology was proposed to be mediated by cues released from astrocytes. Utilizing time-lapse video microscopy in co-cultures of *human* fetal microglial cells and astrocytic cells, the chemokines MIP-1α and MCP-1 were identified as regulators of microglial motility and differentiation [73].

The overexpression of *miR-124* in microglia accelerated the transformation of these cells to an inactivated state through inhibition of the C/EBP-α and PU.1, while the depletion of *miR-124* led to microglial activation both in vitro and in vivo. These findings underscored the potential role of *miR-124* as a regulator of microglial surveillance in the CNS [74]. Microglial polarization is regulated by ARID1A, an epigenetic subunit of the SWI/SNF chromatin-remodeling complex, through alterations of the chromatin state in microglia [75,76]. The migration of microglial cells also seemed to be affected by PGRN, because its knockdown resulted in a failure of microglial precursors to colonize the embryonic retina [77]. The absence of integrin αVβ8 from the CNS prevents microglial transition from immature precursors to a mature state. As αVβ8 controls TGFβ signaling to microglia, these “dysmature” microglial populations are expanded as a consequence of impaired TGFβ signaling during the perinatal period, leading to disrupted oligodendrocyte development, interneuron loss, and neuromotor dysfunction [78]. Epigenetic factors may also affect microglial development. Embryonic HDAC1 and HDAC2 absence disrupts microgliogenesis, altering the crucial acetylation marks implicated in morphology, reactivity, cell cycle, and apoptosis. Specifically, reduced proliferation and induced apoptosis were observed after ablation of the above epigenetic regulators, resulting in the hyperacetylation of specific pro-apoptotic and cell cycle genes [79].

Fate-mapping strategies remain the best way to track cells from the embryonic YS (microglia) versus bone-marrow (monocyte-derived macrophages). In terms of markers, the exact distinction between microglia and periphery-originated macrophages is challenging as they express common markers such as CD11b, CX3CR1, CD45, F4/80, and IBA-1 [80]. Nevertheless, TMEM119 has been recognized as a trans-membranous molecule that is abundantly produced only by microglia, along with P2RY12, but both markers can be downregulated in disease [5,81,82]. However, recently it was proposed that TMEM119 is neither a specific nor a reliable marker for microglial cells [83]. Siglec-H was also found to be a specific marker for microglia in *rodents*, as it was almost absent in CNS-infiltrating monocytes and CNS-associated macrophages [84]. Recently, HexB has also emerged as a promising marker, but the characterization is still largely lacking [85]. On the contrary, CD44 and CD169 are markers expressed only in peripheral-divided cells and not on resident microglia [86,87].

TREM2, as a protein involved in intracellular signals, interacts with transmembrane protein DAP12, thus activating the Wnt/β-catenin pathway and stabilizing β-catenin via blocking GSK3β activation. Thus, TREM2 promotes the survival and proliferation of primary microglial cells [88]. In addition, the transcription factor MAFB may be involved in regulating microglial cell development and homeostasis [89]. The homeostasis is further preserved by the epigenetic regulator MECP2, which controls microglial responsiveness to external stimuli [90,91]. In the postnatal developing brain, the absence of microglial EED, a Polycomb protein vital for synaptic pruning, led to the upregulation of phagocytosis-related genes [92]. Contrariwise, the deletion of microglial *Tgm2* in *mice* resulted in the downregulation of microglial phagocytic-related genes accompanied by synaptic pruning and cognitive impairment [93]. A P2RX7-induced proliferation of embryonic spinal cord microglia was proposed after comparison of wild-type and *P2rx7*-/- embryos. The ablation of *P2rx7* also affected microglial density, while *Pannexin-1*-/- embryos showed unaltered proliferation rates. Altogether, microglial proliferation may be regulated by P2RX7 receptors in a Pannexin-1-independent way during early development [94].

Another in vitro study confirmed that IL-33, which is released by astrocytes and endothelial cells, enhances the proliferation of microglial populations [95]. Similarly, in the uninjured CNS, G-CSF increased microglial numbers [96]. However, the GM-CSF was a stronger stimulus for microglial proliferation in *human* brain cultures [97]. The increasing microglial populations were correlated with a direct effect of GM-CSF upon treatment with IL-5, whereas IL-5 induced an intense cellular metabolism in contrast with GM-CSF treatment in microglial cell cultures [98]. Moreover, 1 ng/mL of CCL-1 mediated chemotaxis, while 100 ng/mL increased motility, proliferation, and phagocytosis of microglial cells in culture [99]. An induction of microglial cell proliferation was mediated in vitro by CCL2 along with *miR-10* [100]. Neurotrophins have a potential role in modulating the proliferation and survival of microglial populations in vitro. Specifically, NGF and BDNF increased microglial proliferation, contrary to NT-3 and NT-4 [101]. Lastly, SCF was identified as a promoter of microglial cell proliferation, migration, and phagocytosis in culture (Table 1) [102].

Summarizing, microgliogenesis is a complex biological process strictly regulated by multiple molecular drivers in a similar pattern to other CNS cells, such as oligodendrocytes [103].

## 4. Spatiotemporal Distribution in Various Species

In *rodents*, microglia were observed in the fetal forebrain at E11, when the telencephalic vesicles form [108]. Other studies identified E12 as the initial point of brain colonization [109,110]. Using in vivo immunohistochemistry and ex vivo time-lapse analysis of microglia, E11.5 was identified as the first day of the microglial entrance in the cortex [111]. The route includes in turn the pial surface, lateral ventricle, and cortical wall, moving over towards the cortical plate in the later embryonic phases. Three invasion phases in the cortical parenchyma have been proposed: (a) between E10.5 and E14.5, a gradual increase in the number of microglial cells takes place, succeeded by (b) a rapid phase with a significant rise in microglia from E14.5 to E15.5, followed by (c) the last slow wave of entry from E15.5 to E17.5. Before the invasion in the parenchyma, the peripheral microglia proliferates, especially at early phases [111]. Stremmel et al. demonstrated that, from E8.5, the CX_3_CR1^+^ pre-macrophages were detectable in the YS proliferating and preparing to enter the blood circulation for their migration to the brain parenchyma, while Kierdorf et al. suggested that E9.5 is the starting point for the migration of microglial progenitors into the neural tube [50]. The invading wave of YS progenitors to the tissue peaks around E10.5, then excessively decreases towards E12.5 and disappears at E14.5. Consequently, microglial progenitors are dependent on the vascular system for their migration [112]. Finally, the transformation of immature microglia into ramified, mature cells occurs between the second and third postnatal week (Figure 2) [113,114].

In *humans*, well-differentiated microglia were observed after 35 weeks of gestation (GW) [115]. However, Rezaie and Male suggested that colonization of the spinal cord starts around 9 GW, with the major influx of microglial cell populations estimated around 16 GW. In the second trimester, the cerebrum is colonized by microglial populations widely dispersed in the intermediate zone at 20–22 GW [116]. In the initial phase of microglial colonization between 12 and 14 GW, two cell populations were identified by Rezaie et al., namely CD68^++^ RCA-1^+^ MHC II^-^ amoeboid cells located in the subplate and RCA-1^++^ CD68^-^ MHC II^-^ progenitors first observed in the marginal layer and lower cortical plate and which ramified within the subplate [117]. In 2006, the first intracerebral microglial populations were described close to the meninges and choroid plexus, next to the di-telencephalic fissure at 5.5 GW, whereas the cortical anlagen was populated with cells starting at 10–12 GW [118]. Routes of entry were found to be different for the cerebral cortex compared with the diencephalon. Microglial cells invaded the cerebrum from the ventricular lumen and the leptomeninges, starting at 4.5 GW. From 12 GW, the intraparenchymal vascular route of entry could be determined [119]. In 2010, Verney et al. suggested that the invasion of amoeboid microglia occurred between 4.5 and 5.5 GW into the *human* forebrain; this is in accordance with the data from other animal models such as *rodents*, regarding the spatiotemporal patterns observed for microglial development. Ultimately, the meninges, choroid plexus, and ventricles were identified as the three early routes of microglial entry [120].

In *avians*, the first microglial population was found to be located within the brain independently of vascularization, reaching the nervous system parenchyma by passing through the pial basal lamina [121]. More specifically, before E9, the cerebellar anlage contained only a small number of microglial precursors. Microglial precursors cross the pial surface at the basal region of the peduncles to enter the cerebellar anlage. Then, microglia proceed radially to the various cortical layers by migrating via the white matter. Following the ultimate settlement of microglial cells, differentiation then ensues [122].

## 5. Proliferation in the Adult Compromised CNS

As the BBB and microglial cell maturation are established, the question arises as to how microglia are renewed in the adult brain. The participation of bone marrow-derived cells in the repopulation of microglial cell niches was proposed in various conditions, especially after bone marrow transplantation [123,124,125,126,127,128], and in diseases such as stroke [129], cerebral ischemia [130], bacterial meningitis [131], entorhinal cortex lesions [132], Parkinson’s disease [133], Alzheimer’s disease [134,135], multiple sclerosis [136], facial nerve axotomy and autoimmune encephalitis [137], scrapie [138], and brain and peripheral nerve injury [139,140,141]. During aging and the transition from plasticity to proinflammatory activation in primary neurodegeneration, the latest data also suggest that many metabolic byproducts and mitochondrial components can serve as damage-associated molecules, creating an extracellular gradient and accumulation of reactive oxygen species, which in turn propagate the inflammatory neurodegeneration [142,143]. Under acute situations such as when a stab wound inflicts damage to a brain region, the resident microglia need the contribution of circulating monocytes to efficiently respond to the extra load of detritus [144]. It has been suggested that even after recovering from severe brain inflammation, resident microglia form a remarkably stable cell pool that is seldom replenished by hematogenous cells in adult animals [145].

A physiological process that aids in the development of the adult microglial cell population is the proliferation of microglial precursors in the developing brain [146]. Lawson et al. suggested that resident microglia synthesize DNA and go on to divide in situ. Additionally, cells were found to be recruited from the circulating monocyte pool through an intact BBB and rapidly differentiated into resident microglia. These two processes contributed almost equally to the steady-state turnover of resident microglia [147]. In a *mouse* model of ALS, the local proliferation of resident microglia had the greatest contribution to the observing microgliosis, while the effects of bone marrow-derived cells were limited among the microglia populations [148]. Strong evidence for the local self-renewal of CNS microglia as the main source of repopulation of adult microglia were obtained from a model using chimeric animals obtained by parabiosis showing that these cells could be maintained independently from bone marrow–derived cells during adulthood in ALS and facial nerve axotomy [149]. However, Ly-6C^hi^CCR2^+^ monocytes were found to be recruited to the lesioned brain differentiating into mature microglia. Remarkably, monocyte invasion during CNS pathology with an intact BBB or in non-diseased adult CNS required previous conditioning of brains, such as direct tissue irradiation [150]. Indeed, brain conditioning with lethal irradiation and alkylating agents such as busulfan was found to be vital for an efficient microglial cell repopulation after hematopoietic stem cell transplantation [151].

In 2013, Li et al. observed that after ischemic stroke, a small number of blood-derived CX3CR1^GFP/+^ cells invaded the brain parenchyma; however, these cells were phenotypically different from resident microglia with distinct kinetics. This study delineated the greatest impact of local resident microglia on the repopulation of parenchymal cells compared to recruited blood-derived cells after ischemic stroke [152]. The efficiency of microglia for self-renewal arising from a CNS-resident pool independently from peripheral myeloid cells was also supported by another experimental study that investigated the repopulation of brain parenchyma using a model of conditional depletion of microglial cells [153]. During the process of cellular restoration, the proliferation of local microglia was found to be dependent on the IL-1 receptor, which was highly expressed by local cell pools. Bone-marrow-derived macrophages populated the brain only after irradiation and bone marrow transplantation, and did not express the IL-1 receptor [153].

In *zebrafish*, using temporal-spatial resolution fate mapping analysis, embryonic microglia emerged from the rostral blood island in a RUNX1-independent and PU.1-dependent manner, while adult microglia originated from the ventral wall of the dorsal aorta in a RUNX1-dependent, c-MYB- and PU.1-independent manner [154]. The microglial self-renewal was shown to resemble a stochastic process at steady state, whereas clonal microglial expansion seems to predominate under unilateral facial nerve axotomy [155]. In another study, the partial microglial depletion resulted in the engraftment of peripherally derived macrophages independently of irradiation. These newly-engrafted cell populations differ transcriptionally from microglia [156]. Similarly, another depletion study showed that the microglial niche is filled with new cells via local proliferation of CX3CR1^+^F4/80^low^Clec12a^–^ microglia and invasion of CX3CR1^+^F4/80^hi^Clec12a^+^ macrophages derived from Ly6C^hi^ monocytes. This engraftment was associated with vascular activation and type I interferon, while it was shown to be independent of BBB integrity [157]. These peripherally engrafted cells were transcriptionally distinct from microglia, showcasing different surface marker expression, phagocytic capacity, and cytokine release [157,158].

Through additional studies, Huang et al. delineated that repopulated microglial cell populations are entirely generated from residual microglial proliferation after acute depletion [159], instead of nestin-expressing progenitors, as was argued in a CSF1R inhibitor-mediated experiment [160]. In agreement with the previous statement, Zhan et al. demonstrated that after acute ablation, the newborn adult microglia generated via self-renewal from the local CX3CR1^+^ microglia without any contribution of nestin^+^ progenitors or peripheral myeloid cells. The repopulated microglia formed stable and distinct clusters with minimum migration capacity via clonal expansion. Although these regenerated microglial cells were presented in an immature state, microglial differentiation was mediated by NF-κB and interferon pathways [161]. A fate mapping study from Chen et al. showed that after neonatal stroke, a monocyte-to-microglia transition is possible [57]. In contrast, a study conducted in 2021 showed that microglia are not replaced by bone-marrow-derived cells in Alzheimer’s disease similar to the BAMs, which seldom replenished the microglial cell pool [162]. Ultimately, microglial cell manipulation is being intensely investigated in the context of immune-mediated diseases such as multiple sclerosis, where microglia are heavily implicated as pathogenic mediators of progressive disease [163,164,165], and targeted therapies are being developed [166,167].

Summarizing the results of the above studies, it is postulated that the greatest contribution to microglial repopulation is based upon its local self-renewal, both in steady state and disease. However, circulating monocytes may also contribute to a lesser extent, especially in disease. The final confirmation of the exact repopulation pattern necessitates further investigation.

## 6. Conclusions

The widely accepted, contemporary view of the origin of CNS-resident microglia is the YS. However, this was hotly debated until the early 2010s. Although this may pass unnoticed to the majority of the research community in the immune-related neuroscience field, understanding the underlying molecular development they undergo during embryogenesis may aid towards developing novel therapies that ideally could decelerate, halt, or reverse neurodegeneration by targeting the microglia-mediated repair process. A main challenge now is to elucidate the precise biological identity of each different microglial state as well as the variable microglial activity per CNS region, allowing us to perform selective interventions. Another field of application that can potentially benefit from relevant developmental research is aging, where mechanisms implicating microgliogenesis can be exploited in favor of slowing the senescent progress by, e.g., combating oxidative stress. Finally, the understanding of cellular ontogeny may enable successful lab-approached manipulations aimed at depletion of microglial cells and beneficial microglial renewal in the CNS, in both homeostasis and disorders.

## Figures and Tables

**Table 1:** Molecular drivers of microglial early differentiation, migration, proliferation, and terminal differentiation.

Gene	Locus	Protein	Species	Biological Role	Ref.
BDNF	11p14.1	Brain derived neurotrophic factor	Mice	Proliferation	[101]
CCL1	17q12	C-C motif chemokine ligand 1	Mice	Migration; Proliferation	[99]
CCL2	17q12	C-C motif chemokine ligand 2	Human; Mice	Migration; Proliferation; Terminal differentiation	[73,100]
CCL3	17q12	C-C motif chemokine ligand 3	Human	Migration; Terminal differentiation	[73]
CSF1	1p13.3	Colony stimulating factor 1	Mice	Proliferation; Terminal differentiation	[47,68]
CSF1R	5q32	Colony stimulating factor 1 receptor	Mice; Zebrafish	Migration; Terminal differentiation	[47,63]
CX3CL1	16q21	C-X3-C motif chemokine ligand 1	Mice	Migration	[72]
CX3CR1	3p22.2	C-X3-C motif chemokine receptor 1	Mice	Migration	[72]
CXCL12	10q11.21	C-X-C motif chemokine ligand 12	Mice; Rat	Migration; Proliferation	[70,71]
CXCR4	2q22.1	C-X-C motif chemokine receptor 4	Mice; Rat	Migration; Proliferation	[70,71]
CXCR7	2q37.3	C-X-C chemokine receptor type 7	Rat	Migration; Proliferation	[71]
DAP12	19q13.12	DNAX-activating protein of 12 kDa	Mice	Proliferation; Terminal differentiation	[67,68]
G-CSF	17q21.1	Granulocyte colony-stimulating factor	Mice	Proliferation	[96]
GM-CSF	5q31.1	Granulocyte-macrophage colony-stimulating factor	Human	Proliferation	[97]
HDAC1	1p35.2–p35.1	Histone deacetylase 1	Mice	Proliferation	[79]
HDAC2	6q21	Histone deacetylase 2	Mice	Proliferation	[79]
IBA1	6p21.33	Ionized calcium binding adaptor molecule 1	Mice	Terminal differentiation	[104]
IL33	9p24.1	Interleukin 33	Mice	Proliferation	[95]
IL34	16q22.1	Interleukin 34	Mice; Zebrafish	Migration; Terminal differentiation	[47,63]
IL5	5q31.1	Interleukin 5	Rat	Proliferation	[98]
INOS	19p13.11	Inducible nitric oxide synthase	Mice	Proliferation	[105]
IRF8	16q24.1	Interferon regulatory factor 8	Mice	Early differentiation	[50]
ITGAV	2q32.1	Integrin subunit alpha V	Mice	Terminal differentiation	[78]
ITGB8	7p21.1	Integrin subunit beta 8	Mice	Terminal differentiation	[78]
MAFB	20q12	MAF bZIP transcription factor B	Mice	Terminal differentiation	[89]
MEIS3	19q13.32	Meis homeobox 3	Mice	Terminal differentiation	[66]
MIF	22q11.23	Macrophage migration inhibitory factor	Mice	Proliferation	[70]
MMP8	11q22.2	Matrix metallopeptidase 8	Mice	Migration	[50]
MMP9	20q13.12	Matrix metallopeptidase 9	Mice	Migration	[50]
NGF	1p13.2	Nerve growth factor	Mice	Proliferation	[101]
NOX2	Xp21.1-p11.4	NADPH oxidase 2	Mice	Migration	[69]
P2RX7	12q24.31	Purinergic receptor P2X 7	Mice	Proliferation	[94]
PGRN	17q21.31	Progranulin	Zebrafish	Migration	[77]
RUNX1	21q22.12	RUNX family transcription factor 1	Mice	Proliferation; Early and terminal differentiation	[59,60]
SALL1	16q12.1	Spalt like transcription factor 1	Mice	Terminal differentiation	[66]
SALL3	18q23	Spalt like transcription factor 3	Mice	Terminal differentiation	[66]
SCF	12q21.32	Stem cell factor	Mice	Migration; Proliferation	[102]
SPI1	11p11.2	Transcription factor PU.1	Mice	Early differentiation	[50]
TGFB1	19q13.2	Transforming growth factor beta 1	Mice	Terminal differentiation	[65]
TREM2	6p21.1	Triggering receptor expressed on myeloid cells 2	Mice	Proliferation	[88]
VEGFR1	13q12.3	Vascular endothelial growth factor receptor 1	Mice	Migration	[69]

Data are retrieved from “The Human Protein Atlas” [106], and “Gene” database of the National Center for Biotechnology Information [107]. Ref.: references.

## Data Availability

Not applicable.

## Abbreviations

AGM	Aorta-gonad-mesonephros
Akt	Protein kinase B
ALS	Amyotrophic lateral sclerosis
ARID1A	AT-rich interaction domain 1A
BAMs	Border-associated macrophages
BATF3	Basic leucine zipper transcriptional factor ATF-like 3
BBB	Blood–brain barrier
BDNF	Brain-derived neurotrophic factor
C/EBP-α	CCAAT/enhancer-binding protein alpha
CCL1	Chemokine (C-C motif) ligand 1
CCL2	Chemokine (C-C motif) ligand 2
CCL3	Chemokine (C-C motif) ligand 3
CCR2	C-C chemokine receptor type 2
CD11b	Cluster of differentiation molecule 11B
CD206	Cluster of differentiation molecule 206
CD45	Cluster of differentiation molecule 45
CD64	Cluster of differentiation molecule 64
CD68	Cluster of differentiation molecule 68
CNS	Central nervous system
CSF-1	Colony stimulating factor-1
CSF1R	Colony stimulating factor 1 receptor
CX3CL1	Chemokine (C-X3-C motif) ligand 1
CX3CR1	CX3C motif chemokine receptor 1
CXCL12	C-X-C motif chemokine ligand 12
CXCR4	C-X-C chemokine receptor type 4
CXCR7	C-X-C chemokine receptor type 7
DAP12	DNAX-activating protein of 12 kDa
E	Embryonic day
EED	Embryonic ectoderm development
EMPs	Erythro-myeloid progenitors
Erk1/2	Extracellular signal-regulated kinase 1/2
G-CSF	Granulocyte colony stimulating factor
G-CSFR	Granulocyte colony stimulating factor receptor
GM-CSF	Granulocyte-macrophage colony-stimulating factor
GM-CSFR	Granulocyte-macrophage colony-stimulating factor receptor
GSK3β	Glycogen synthase kinase-3 beta
GW	Gestational week
HDAC1	Histone Deacetylase 1
HDAC2	Histone Deacetylase 2
HOXB8	Homeobox B8
HSC	Hematopoietic stem cells
IBA1	Ionized calcium binding adaptor molecule 1
ID2	Inhibitor of DNA binding 2
IL-33	Interleukin 33
IL-34	Interleukin 34
IL-5	Interleukin 5
iNOS	Inducible nitric oxide synthase
IRF8	Interferon regulatory factor 8
KLF4	Krüppel-like factor 4
M-CSF	Macrophage colony-stimulating factor
M-CSFR	Macrophage colony-stimulating factor receptor
MAFB	MAF bZIP transcription factor B
MCP-1	Monocyte chemoattractant protein-1
MECP2	Methyl-CpG binding protein 2
MEIS3	Meis homeobox 3
MHC II	Major histocompatibility complex class II
MIF	Macrophage migration inhibitory factor
MIP-1α	Macrophage inflammatory protein-1 alpha
miR-124	microRNA 124
MMP8	Matrix Metallopeptidase 8
MMP9	Matrix Metallopeptidase 9
MPO	Myeloperoxidase
MYB	MYB proto-oncogene, transcription factor
NF-kB	Nuclear factor kappa B
NGF	Nerve growth factor
NOX2	NADPH oxidase 2
NR4A1	Nuclear receptor subfamily 4 group A member 1
NT-3	Neurotrophin-3
NT-4	Neurotrophin-4
P2RX7	P2X purinoceptor 7
P2RY12	Purinergic receptor P2Y12
PGRN	Progranulin
qRT-PCR	Quantitative reverse transcriptase polymerase chain reaction
RCA-1	Ricinus communis agglutinin-1
RUNX1	Runt-related transcription factor 1
SALL1	Spalt like transcription factor 1
SALL3	Spalt like transcription factor 3
SCF	Stem cell factor
SVZ	Subventricular zone
TGF-β1	Transforming growth factor beta 1
TGM2	Transglutaminase 2
TMEM119	Transmembrane Protein 119
TREM2	Triggering receptor expressed on myeloid cells 2
VEGFR1	Vascular endothelial growth factor receptor-1
YS	Yolk sac
αVβ8 integrin	Integrin subunit alpha V and beta 8
